# Dr. James E. Garretson

**Published:** 1896-01

**Authors:** 


### Obituary.

### DR. JAMES E. GARRETSON.

JAMES EDMUND GARRETSON, A. M., M. D., D. D. S., professor of anatomy and surgery in the Philadelphia Dental College and dean of that institution, died at his home in Lansdowne, Pa., on the evening of Saturday, October, 26, 1895.

Dr. Garretson was born in Wilmington, Delaware, October 18, 1828, and commenced the study of dentistry with Dr. Thacher, of Wilmington, in 1850. He began practice in the neighborhood of Woodbury, N. J., and later came to Philadelphia, where he entered as a student in the Philadelphia College of Dental Surgery, the progenitor of the present Pennsylvania College of Dental Surgery. He received his degree in dentistry on February 29, 1856, and entered upon the practice of his profession in Philadelphia. Among his classmates was his brother-in law, Dr. J. Foster Flagg, for many years his colleague in the faculty of the Philadelphia Dental College.

He began the study of medicine at the University of Pennsylvania, graduating from that institution in 1859. The same year he was married to Beulah, a daughter of George Craft, of Upper Greenwich, N. J. At the organization of the Philadelphia Dental College in 1862, Dr. Garretson became a member of the faculty as professor of pathology and therapeutics, but before delivering his course of lectures he resigned to accept the position made vacant by resignation of the late Professor D. Hayes Agnew in the Philadelphia School of Anatomy, in which school Dr. Garretson had been a demonstrator for five years. He continued in charge of the school until 1864, when he gave up that practice and entered the Philadelphia Dental College as professor of anatomy and surgery. During the war of the rebellion Dr. Garretson for a time was in active military hospital service. In 1869 he was appointed oral surgeon to the hospital of the University of

Pennsylvania. In 1880 he became dean of the Philadelphia Dental College, which position he faithfully and acceptably filled until his death.

In the special field of his activities Dr. Garretson filled a unique place. He was the pioneer in a new department of surgery, and the creator of its technique. He brought to the practice of his life-work the skill and manual dexterity of the trained dentist, to which was added the broad culture and intimate knowledge of his subject required by the educated surgeon. With this educational equipment grafted upon his rich natural endowment of attractive characteristics, a combination resulted which easily accounts for his phenomenal success and wide reputation as a surgeon and as a teacher.

Broadly speaking, Dr. Garretson may be said to be a striking example of the self-made man. His love for his work, his faith in and respect for the possibilities of development in dentistry, and his ambition to secure for it the status and recognition it deserved, have borne abundant fruit in the example of success which he has left as a heritage to his profession.

He realized the crudity which characterized the method of performance of the earlier operations done upon the head, face and jaws; he saw that the special training and many of the operative methods of the dentist were, with suitable modification, directly applicable to surgery within his selected territory. Grasping this great principle and putting it to thorough practical test, he soon found that he had struck the keynote to success. The whole complexion of his operations, whether viewed as to their technique or as to the character of the results, differed essentially from the work done by any of his predecessors and the majority of his contemporaries. His work was distinctly conservative in character. He kept in view the importance of the cosmetic feature of his results, and as a consequence his operations were designed and done with a distinct purpose to avoid to the utmost extent permanent mutilation.

It was his custom as far as possible in operations upon the jaws to perform them within the mouth in order to avoid external incisions, and he carried the development of this principle to the extent that he frequently operated for the removal of the entire superior maxilla through the mouth without external incision. His conservatism was further manifested in respect to the several tissues of the oral cavity, it being a cardinal principle with him never to remove healthy tissue that might in any degree help to bring about a normal restoration of function within the territory of operation. His operations upon the inferior maxilla always involved this feature so far as conditions permitted: Hence it was his custom to leave a thin basilar rim of bone and periosteum for inducing the reformation of a maxillary ridge to be utilized as a base of support for an artificial crown.

His success in operations of this character is in strong contrast with the results shown by the general surgeon who lacks the familiarity with dental methods and requirements possessed by Dr. Garretson.

The record of his results would be incomplete without a reference to his use of the dento-surgical engine in his operative procedures. That he should at once appreciate the applicability of the engine to bone operations was the natural consequence of his dental training. He was among the earliest, if not the first, to successfully make use of this very important and, in many operations, essential aid. He kept it in constant service, and its efficiency is everywhere reflected in the superior results attained by him in his operations, as well as in the performance of certain operations—especially those within the brain-case—that would be impossible for performance without it. The practical development of the surgical uses of the engine is inseparably connected with the record of his surgical work.

The permanent record of his surgical work is embodied in his greatest literary work, the "System of Oral Surgery." This book has passed through six editions, the first appearing in 1869 and the last in 1895.

It is in the last edition of this great work that the culmination of his endeavor to place dental surgery upon a parity with the officially recognized medical specialties is seen. The former editions exhibit the gradual evolution of a professional condition which began in toleration and has since grown into a just recognition.

Dr. Garretson's intellectual qualities were strongly characteristic. His pronounced penchant for philosophical and metaphysical study was one of the determining features of his life. This love for philosophical study manifested itself strongly in his writings, and in his lectures to his students. He possessed wonderful insight into the profounder psychic problems of life, and great facility in their interpretation and portrays to others. His fluency as a speaker, his intense appreciation of the divinity which doth hedge a man, his kindliness and sympathetic quality, often gave to his formal lectures and addresses an oratorical quality of high grade.

Dr. Garretson was a contributor not only to the literature of his profession, but to general literature as well. The earliest of his writings, so far as we have been able to trace, are a contribution upon "Dental Hygiene," and one upon the "Ether Question," published in the *Dental News Letter* in 1855. His writings consisted of a number of separate book publications, as well as of contributions to periodical literature. There have appeared in the *Dental Cosmos* upward of one hundred articles from his pen, besides a large number of unsigned communications published as Hints and Queries.

Apart from his "System of Oral Surgery," he has published works as follows: "Brushland," "Hours with John Darby," "Thinking and Thinkers," "Odd Hours of a Physician," "Nineteenth Century Sense," and "Man and His World." These were written under the *nom de plume* of "John Darby."

Dr. Garretson possessed to a marked degree attributes of simplicity and guilelessness. He had a childish faith in the honesty of mankind; years never taught him even the shadow of a distrust or suspicion of his fellowman, which might have spared him much of the care and anxiety which clouded his last days.

The cause of his death was stated by his physician to be enteritis; many of his friends, however, believe that his death was hastened, if not induced, by mental anxiety caused by the difficulties resulting in the recent separation between the Philadelphia Dental College and the Medico-Chirurgical College. His death was preceded by profound nervous exhaustion, which increased to the end.

In accordance with his earnestiy expressed wish, his remains were incinerated at the Germantown Crematory, on Wednesday, October 30, and the ashes subsequently interred at the Friends' burying ground in Upper Darby—Dr. Garretson being a member of that faith.

The surviving members of hłs family are his widow and two daughters.—*Dental Cosmos.*

